# Bio-imaging and Photodynamic Therapy with Tetra Sulphonatophenyl Porphyrin (TSPP)-TiO_2_ Nanowhiskers: New Approaches in Rheumatoid Arthritis Theranostics

**DOI:** 10.1038/srep11518

**Published:** 2015-07-08

**Authors:** Chunqiu Zhao, Fawad Ur Rehman, Yanlong Yang, Xiaoqi Li, Dong Zhang, Hui Jiang, Matthias Selke, Xuemei Wang, Chongyang Liu

**Affiliations:** 1 State Key Laboratory of Bioelectronics, School of Biological Science and Medical Engineering, Southeast University, Nanjing 210096, China; 2 Department of Chemistry and Biochemistry, California State University, Los Angeles, CA 90032, USA; 3 Daping Hospital, Third Military Medical University, Chongqing, 400042 P.R. China; 4 NanJing Foreign Language School, Nanjing 210096, China

## Abstract

Since Rheumatoid arthritis (RA) is one of the major human joint diseases with unknown etiology, the early diagnosis and treatment of RA remains a challenge. In this contribution we have explored the possibility to utilize novel nanocomposites of tetera suplhonatophenyl porphyrin (TSPP) with titanium dioxide (TiO_2_) nanowhiskers (TP) as effective bio-imaging and photodynamic therapeutic (PDT) agent for RA theranostics. Our observations demonstrate that TP solution PDT have an ameliorating effect on the RA by decreasing significantly the IL-17 and TNF-α level in blood serum and fluorescent imaging could enable us to diagnose the disease in subclinical stages and bio-mark the RA insulted joint.

Rheumatoid Arthritis (RA) is one of the major human progressive joint inflammatory diseases affecting one percent of population^1^. It is considered to be an autoimmune disease with multiple triggers. Susceptibility to RA is also a function of the gender, lifestyle and genotype of the individual. The RA prevalence ratio is higher in female than male individuals and smokers are more prone to be affected by RA than nonsmokers^2^. RA is primarily a disease of the joints along with involvement of other systems including skin and internal organs. Due to its long term and progressive nature, after 10–15 years of progression of the disease 50–70% patients have significant loss in functionality^3^. To date, early diagnosis and treatment of RA remains a major challenge. There is no proper treatment available and only symptomatic treatment with the TNF-α blockers, azathioprine, minocycline, and Non-Steroidal Anti-inflammatory Drugs (NSAID) are given to suppress the symptoms of the disease^4^. It is known that the inflammation is a cellular and vascular response to a stimulus, involving Neutrophils, Macrophages, Lymphocytes, Natural killer cells, dendritic cells, endothelial cells and cell mediators, including interleukins (IL-1, IL-2, IL-6, IL-10, IL-17), tumor necrosis factor alpha (TNF-α), interferons (INFγ), prostaglandins, histamines and serotonin^5^. The primary function of macrophages is to present antigen^6^. In case of RA, in the synovial milieu fibroblasts start function like antigen presenting cells. Hence, fibroblasts invite various inflammatory cells and antibodies to the site, although their primary role is to repair damaged tissue^7^,^8^. Meanwhile, TNF-α is also considered to be one of the key factors in the initiation of inflammatory process in RA^5^. TNF- α can degrade the synovial membrane and initiate the bone resorption by activating chondrocytes and osteoclasts, respectively^9^. Thus, TNF-α has been reported as a potent angiogenic factor in synovial membrane of RA patients^10^. On the other hand, the interleukin 17 (IL-17) is secreted by the CD4^+^ T lymphocyte cells and considered to be a very important pro-inflammatory factor in initiation of inflammation. Recently, the key role of IL-17 in the RA synovial milieu has been explored. It is frequently secreted by the RA synovial cultures *in vitro* as well as *in vivo*^11^,^12^. IL-17 has a vital role in bone resorption along with TNF-α and also mimics the osteoclast differentiation^13^. Therefore, blocking or decreasing the concentration both TNF-α and IL-17 can help to ameliorate RA patients.

Photodynamic therapy (PDT) as a potential therapy against tumors, Rheumatoid arthritis (RA), skin diseases and microbial infections has been explored for the last few decades. PDT consists of a Photosensitizer (PS) (i.e., a porphyrin derivative), molecular oxygen and external visible light^14^. The Photosensitizer is usually injected into the tumor or infection site and excited by light (visible range) in the presence of molecular oxygen, which produces singlet oxygen (^1^O_2_), and possibly other reactive oxygen species (ROS)^15^. Singlet oxygen oxidizes DNA (mainly guanine) as well as cellular organelle membranes and interfere with cellular signal pathways leading to necrosis or apoptosis of the cells in target tissue, e.g., tumor, inflammation or infections^16^. Although some effort has been expanded to explore PDT for treatment of Rheumatoid arthritis, there are as of now no photosensitizers designed specifically for RA therapy. Porphyrin derivatives have been among the most commonly used photosensitizers in PDT^17^. The water-soluble porphyrin Tetra Sulphonatophenyl Porphyrin (TSPP) may have potential application in cancer therapy, and infectious diseases where inflammation is well documented^18^,^19^. By combination with nanoscaled composites^20^, TSPP could potentially be used as diagnostic and therapeutic agent for tumors, and perhaps various other diseases. It has previously been suggested that titanium dioxide (TiO_2_) could be utilized as a photodynamic therapeutic agent in cancer^21^,^22^ and other microbial infections^23^. Nanoporphyrins have recently been reported^24^ as multi-task agents i.e. near infrared fluorescence imaging (NIRFI), positron emission tomography (PET), magnetic resonance imaging (MRI), photo-thermal and dynamic therapeutic agent coined as ‘all in one’. Like other PS, upon exposure to visible light, TiO_2_ can readily produce ROS, both singlet oxygen^25^,^26^ and radicals (OH^-^) by transferring electrons to nearby oxygen containing molecules^27^. TiO_2_ nanoparticles are more biocompatible, i.e., environmentally friendly^28^ and less toxic than other nanoparticles^29^. These properties make nanoscaled TiO_2_ an excellent candidate for biomedical application in various infectious and non-infectious diseases including cancer^30^. On the basis of the above considerations, in this study we have explored the possibility of the potential application of TSPP in combination with TiO_2_ nanowhiskers as an effective bio-marker and photodynamic therapeutic agent for RA disease theranostics. To the best of our knowledge, there have no previous reports about RA early diagnosis with *in vivo* fluorescent imaging of the Arthritis location and concomitant effective photodynamic therapy for RA.

## Results

Initially, we explored the possibility of potential application of TSPP in combination with TiO_2_ both for RA early diagnosis via *in vivo* fluorescent imaging of the Arthritis location and as a photodynamic therapeutic agent for RA. RA early diagnosis with fluorescent imaging before the onset of clinical signs and an effective therapy could indeed be readily realized through this new strategy. The general body weight gain of rats was satisfactory in TP-0.4 group (treatment group against RA disease with injection of 0.4 ml TiO_2_+TSPP compound) with the mean weight gain 22.09 ± 6.71 grams, as compared to 15.17 ± 4.67 grams in the TP-0 group (control group without injection), during the whole experimental trial.

### Blood analysis

**Figure 1:**
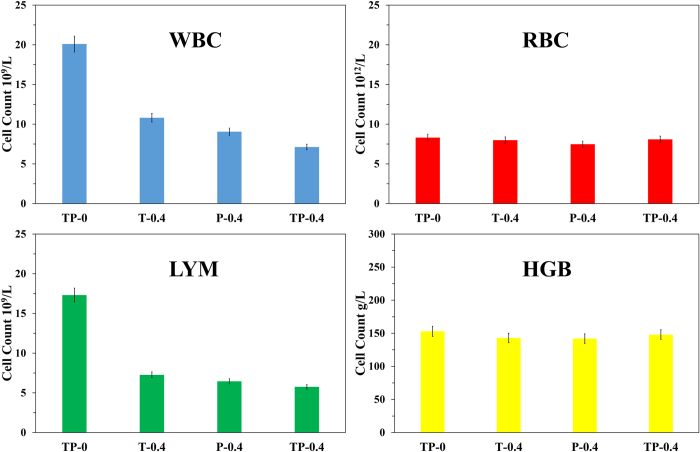
Complete blood cells count affected by TSPP and TiO_2_ as Photodynamic Therapeutic Agents. Here, in this figure shows the CBC results of treatment group TP-0.4, T-0.4, P-0.4 and control group TP-0. Whereas WBC stand for White Blood Cells (10^9^/L), RBC for Red Blood Cells (10^12^/L), LYM for Lymphocytes (10^9^/L), HGB for Hemoglobin (g/L).

Complete blood analysis report indicated that the effect of PDT by TP (TP-0.4) was highly significant (p ≤ 0.01) on the total lymphocytes count and white blood cells when the results were compared with the control group (TP-0) and other treatment group (T-0.4 for TiO_2_ only and P-0.4 for TSPP only). The average WBC count was 7.13 × 10^9^/L with Standard Deviation (SD) of ±0.612 in TP-0.4, 10.81 ± 1.525 (SD) 10^9^/L in T-0.4 group, 9.06 ± 1.751 (SD) 10^9^/L in P-0.4 group and 20.10 ± 2.152 (SD) 10^9^/L in TP-0 group. The mean value for lymphocytes in TP-0.4 group was 5.76 ± 0.230 (SD) 10^9^/L, T-0.4 group was 7.27 ± 0.290 (SD) 10^9^/L, P-0.4 group was 6.45 ± 0.129 (SD) 10^9^/L and TP-0 group was 17.32 ± 0.519 (SD) 10^9^/L. Meanwhile, there was significantly less effect from the PDT of TP-0.4 group on the hemoglobin and red blood cells compared with group P-0.4 (p ≤ 0.01). In TP-0.4 the hemoglobin and RBC mean values were 148.05 ± 5.921 (SD) g/L and 8.09 ± 0.511 (SD) 10^12^/L, respectively, while in TP-0 hemoglobin value was 153.00 ± 4.592 (SD) g/L and the RBC mean value was calculated as 8.32 ± 0.554 (SD) 10^9^/L. But the PDT of P-0.4 group effected both the hemoglobin values (142.00 ± 2.841 (SD) g/L) and RBC mean values (7.49 ± 1.115 (SD) 10^12^/L). Also, no difference for the neutrophils count was found between group TP- 0.4 and TP-0. The mean value of neutrophils was 1.78 ± 0.511 (SD) 10^9^/L and 2.19 ± 0.398 (SD) 10^9^/L in TP-0.4 and TP-0, respectively (see Fig. 1).

### Serum Analysis

**Figure 2:**
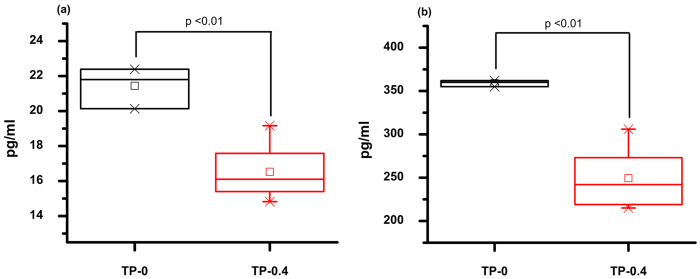
Blood serum level for Interleukin (IL) 17 and Tumor Necrosis factor (TNF-α) in SD rats. In this figure the black box shows the control group TP-0 and red one for the treatment TP-0.4. Fig. 2a shows the concentration level of IL-17 in rats and Fig. 2b represents the Tumor necrosis factor α level in serum. P < 0.01 indicates the highest significance level between the two groups.

The significance level of PDT on IL-17 and TNF-α was very high i.e. (p ≤ 0.01). The average mean IL-17 value calculated was 16.53 ± 1.642 (SD) and 21.84 ± 1.128 (SD) pg/ml in TP-0.4 and TP-0, respectively, as shown in Fig. 2a. The mean value of TNF-α was calculated as 249.38 ± 35.30 (SD) pg/ml in TP-0.4 and 358.69 ± 3.59 (SD) pg/ml in TP-0 group (Fig. 2b).

### Histopathology

**Figure 3:**
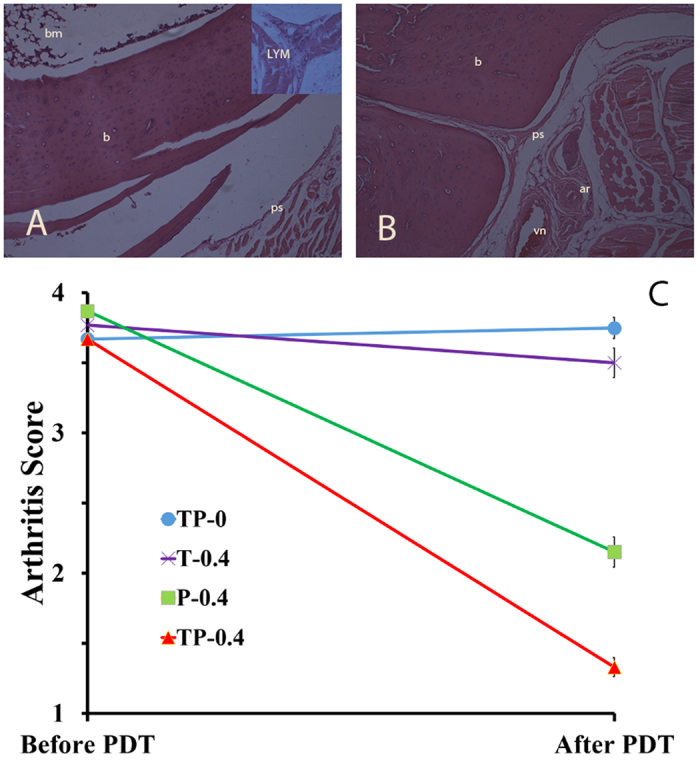
SD rat feet histopathology and Arthritis score of treatment TP-0.4 and control TP-0 group. Here figure A shows TP-0 as control, B shows TP-0.4 treatment group, where bm stands for bone marrow, b for bone, ps for periosteal membrane, LYM for lymphocytes, ar for artery and vn for vein. Figure C shows the Arthritis score with in treatment (TP-0.4, T-0.4 and P-0.4) and control (TP-0) group, the lines explain time duration from the day 0 (first of PDT) to the last day of the experiment.

Histopathology sections of TP-0.4 and TP-0 group are shown in Fig. 3. Histopathology examination of treatment group TP-0.4 revealed less cellular infiltrations, more joint space and less erosion of the synovial membranes compared to the control group TP-0. The ankle joint became narrower in the TP-0 control group and very frequent pannus formation was observed around the periosteal and bone resorption area.

### Arthritis Score

The signs of arthritis and degree of inflammation were calculated according to the procedure mentioned earlier. The average mean calculated before PDT was 3.67 ± 0.516 (SD) for both treatment (TP-0.4, T-0.4, P-0.4) and control (TP-0) groups. After PDT the mean value for TP-0.4 group was 1.33 ± 0.516, whereas for the P-0.4 group the value was 2.15 ± 0.681, while both T-0.4 and TP-0 remained the same with almost no change (Fig. 3C). In TP-0.4 the decrease in edema for left and right foot was 12.8 and 7.64 percent, respectively, whereas in the TP-0 group there was a 15.24 and 16.32 percent increase in the left and right foot, respectively (Movies.S1, S2).

### Fluorescence Imaging

**Figure 4:**
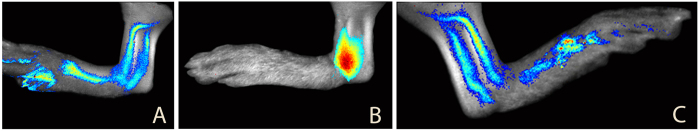
DBA/1 mice in TP-0.4 group showing fluorescence before the onset of clinical signs on the day 16 of collagen-adjuvant injection. In this figure A, B and C shows the fluorescence before the onset of clinical signs and it can be used for detection of rheumatoid arthritis in early development stages.

**Figure 5:**
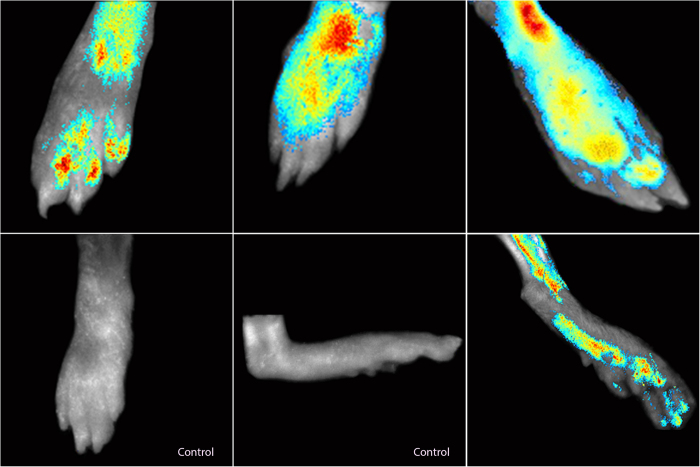
DBA/1 mice in TP-0.4 showing fluorescence on day 28 of collagen-adjuvant injection. In this figure, the control group shows no fluorescence, the treatment group shows fluorescence which also showed before the onset of clinical signs.

**Figure 6:**
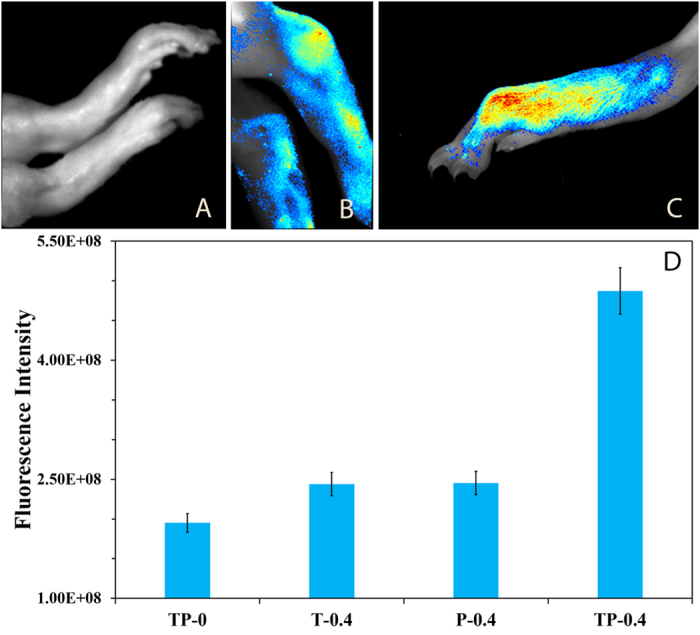
SD rats showing fluorescence in the rheumatoid arthritis joints when treated with TiO_2_ and tetra suplhonatophenyl porphyrin (TSPP). Here A is control TP-0, showing no fluorescence, B is TP-0.4 showing fluorescence at tibia-tarsal joint and C shows fluorescence in the infected foot. Figure D shows the fluorescence intensities of different groups.

For early diagnosis fluorescence imaging was done before the onset of clinical signs. The images from the mice feet in the treatment group showed very strong fluorescence on day 16 (Fig. 4). The same animal showed obvious clinical signs at a later stage and very strong fluorescence upon exposure to green light (500–550 nm) (Fig. 5). The same procedure was repeated for rats, as well (Fig. 6).

**Figure 7:**
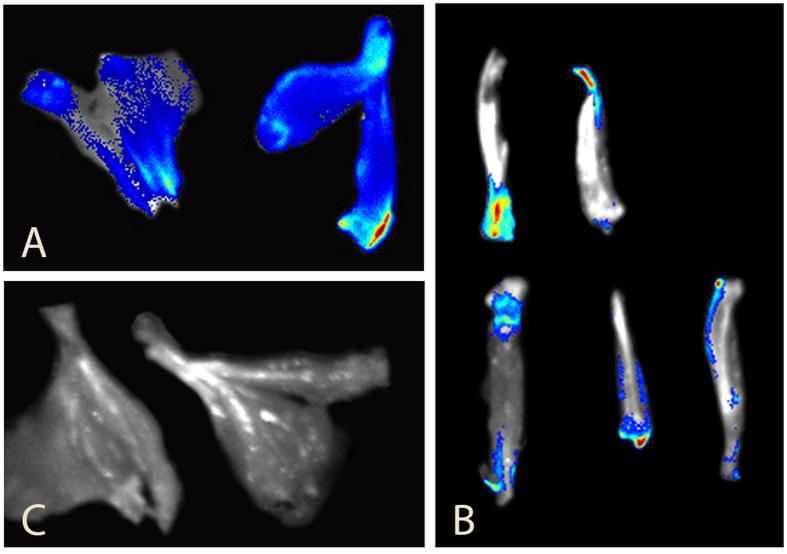
Sagittal section of forelimbs from DBA/1 mice showing fluorescence bio-marking of the affected sites. This figure shows the sagittal section of rear feet in DBA/1 mice after therapy, clearly demonstrating the accumulation of TP in the infected joints by showing intense fluorescence, meanwhile the other parts are without fluorescence; here, A and B shows clear fluorescence in the infected RA joint in the treatment group TP-0.4, whereas C show, no fluorescence in normal group.

**Figure 8:**
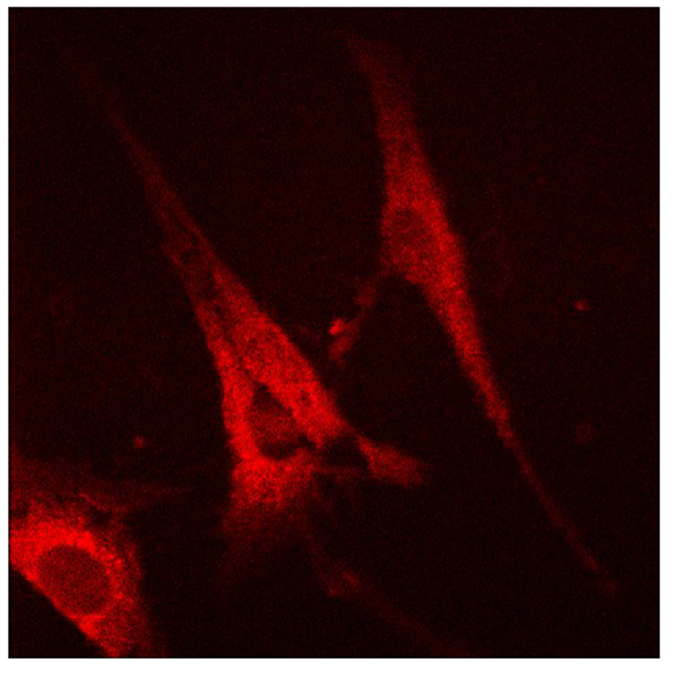
*In vitro* Fibroblast cells from rheumatoid arthritis joint of SD rats showing bright intracellular fluorescence.

To determine the exact location of TP within the foot, the sagittal section of foot was imaged separately for fluorescence microscopy. Interestingly, only the infected joints showed fluorescence and weak fluorescence was found in muscles, suggesting the TP higher concentration residing in insulted tissues (Fig. 7). Moreover when the fibroblast cells cultured from the RA synovium were treated with TP, they showed a very strong fluorescence upon excitation by green light under the confocal microscope, showing the successful absorbance of TP within the cell (Fig. 8).

## Discussion

The above study demonstrates the potential of TSPP in combination with TiO_2_ as a new photodynamic therapeutic agent for RA. Early diagnosis with fluorescent imaging by using TSPP along with TiO_2_ nanowhiskers before the onset of clinical signs and effective therapy were kept in focus. It is well known that nano TiO_2_ has a strong photodynamic therapeutic effect in relevant disease treatment^31^, which also ensured the efficient safe delivery of TSPP macromolecules with TiO_2_ nanowhiskers to the diseased joint synovium.

The etiology of RA is still unknown, and only targeting certain cytokines and decreasing their concentration will reduce its specific symptoms. Cachexia is among the common problems associated with RA, characterized by the body weight loss, debility and muscular wasting in addition to loss of appetite; its etiology may be associated with TNF-α^5^. In our results the general effect of PDT on the weight gain was relatively good and progressive. This increase in growth rate can be attributed to the decrease in TNF-α concentration.

Blood cells and their mediators have very important roles in inflammation and the body immune system. WBC, especially total lymphocytes count was highly decreased by PDT. The lymphocyte cells play very vital role in inflammatory process and auto immunity. Cytokines regulate lymphocytes in the synovium and other inflammatory tissues^32^,^33^. In RA lymphocytes level is always reported in much higher level^34^, but in the case of the TP, photodynamic therapy efficiently lowered that population, which is consistent with that earlier reported by Neupane *et al.*^35^ where Methotrexate were used as PS. In our results the RBC and hemoglobin levels were unchanged and remained the same as for the control. This is due to the synergistic effect of TiO_2_ with TSPP, as earlier studies with only porphyrin as PS suggested that PDT decreased the RBC level and increased the hemoglobin level^36^, where due to toxic effects of porphyrin the lyses of RBC occurs and hemoglobin level in blood were increased.

To confirm that the TSPP-TiO_2_ composite material does still produce singlet oxygen, we conducted time-resolved measurements of the singlet oxygen luminescence of TSPP and TSPP-TiO_2_ (1:10). A plot of singlet oxygen luminescence intensity vs. optical density of the materials is shown in Fig. S1 for TSPP and TSPP-TiO_2_ (1:10). The ratio of the slopes of these plots (0.69) gives the singlet oxygen quantum yield (Φ^Δ^) for TSPP-TiO_2_ (1:10) relative to TSPP. Using the literature value of FD = 0.64 for TSPP^37^, we obtained a singlet oxygen quantum yield of 0.44 for TSPP-TiO_2_ (1:10). Although singlet oxygen production is slightly diminished compared to free TSPP, the composite materials may be more beneficial than the free sensitizers due to the controlled release of TSPP and the decrease of toxic effects on healthy tissues and blood cells (especially on RBC and hemoglobin). Furthermore, the TSPP-TiO_2_ (1:10) material still produces an appreciable amount of ^1^O_2_ to ensure effective a strong therapeutic effect.

Interleukins are a key factor in inviting and maintaining the inflammatory cells within the synovium. IL-17 has a synergistic effect with TNF-α^38^, and in our study we found that both IL-17 and TNF-α level are decreased in the treated model with TP. Most researchers investigated and blocked only IL-17 or TNF-α to decrease the inflammation in RA, with the latter one being more effective.

Increase or decrease in IL-17 can affect the degree of RA, accordingly. Our findings suggest that decrease in the concentration of IL-17 can be attributed to decreases in the degree of inflammation, consistent with earlier reports^39^.

Inflammation is generally dissolved by natural mediators e.g. annexin 1^40^, down regulates the extravasation of leukocytes into the tissue, and lipoxin^41^ inhibit the neutrophils and promote the monocytes immigration to the tissue but at the same time lipoxin also invites the monocytes. In contrast with the case of RA they are gradually replaced by pro inflammatory mediators^8^. The role of fibroblast in the RA synovium is pertinent to mention, fibroblast cells are a group of connective tissue cells mainly responsible for tissue repair during trauma or insult. Nevertheless, ample evidence defines their critical role in RA microenvironment for inviting lymphocyte to the synovium^42^,^43^.

**Figure 9:**
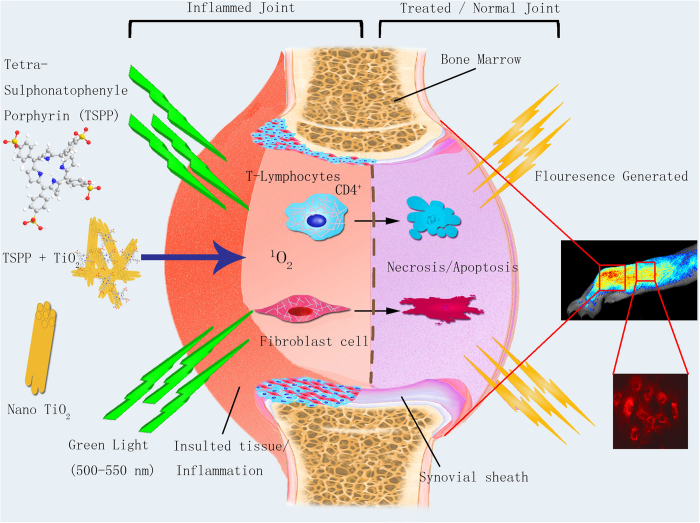
Illustration of bio-imaging and PDT effect with TSPP and TiO_2_ Nanowhiskers on the Rheumatoid Arthritis joint.

Our spectroscopic and electrochemical studies also show significant differences between the interaction of TSPP with the urine collected from treatment group and the normal group without RA disease (Data not shown). It is evident that the urine from treatment group can significantly reduce or smear out the specific absorption or electrochemical signal of TSPP but the urine from normal group without RA disease has almost no effect. These observations suggest that TSPP could possibly be used for the early detection of the inflamed joints of RA disease, consistent with the *in vivo* fluorescence imaging: The fluorescence imaging of murine feet sagittal section clearly demonstrates the localization of higher concentration TSPP and TiO_2_ within the inflamed joints, while the soft tissues showed relatively lower fluorescence intensity. *In vitro* the fibroblast cells cultured from RA synovium also showed strong fluorescence. As shown in Fig. 9, in PDT excitation of the PS (TiO_2_ and TSPP) by visible light in the presence of molecular oxygen leads to the generation of reactive oxygen species, including singlet oxygen (^1^O_2_) which then interferes with cellular pathways of adjacent cells and induce apoptosis or necrosis. The ^1^O_2_ lifespan of singlet oxygen generated within the tissue is ~3 μs^44^. The fluorescence images clearly indicate the localization of TP in the RA synovium and upon exciting with green light (500–550 nm) it produced singlet oxygen to necrotize the local cells, i.e., fibroblasts, lymphocytes, etc. It has also been reported that ROS resides for about eighteen hours in the target tissue^17^, which in case of RA synovium is enough time to induce the apoptosis in resident lymphocyte and fibroblasts.

## Materials and Methods

### Experimental animals

Male SD strain rats and DBA-1 mice were selected to produce Collagen Induced Arthritis (CIA), because they represent excellent model for CIA. All animals were provided with standard pallet food and water at ad-libitum, with a regular 12/24 light cycle. Average weight for rats and mice calculated at the beginning of treatment was 200 ± 10 and 21 ± 0.5 (8 weeks age) grams, respectively. All experiments involving mice were approved by the National Institute of Biological Science and Animal Care Research Advisory Committee of Southeast University, and experiments were conducted following the guidelines of the Animal Research Ethics Board of Southeast University. All the chemicals used in cell culture were purchased from HyClone Laboratories, Inc. 925 West 1800 South Logan, Utah 84321, USA. Chemical used in CIA induction were purchased from Chondrex, Inc. 260715 1st Place NE Redmond, WA 98052.

### Experimental procedure

SD-rats were divided into three groups i.e. a normal group without RA disease, a control group with RA disease (TP-0) and treatment group against RA disease. The normal group and control group were non-injected (i.e., without TP treatment) while the later one was injected (i.e., TP-0.4 group inject 0.4 ml TiO_2_+TSPP compound, T-0.4 group inject 0.4 ml TiO_2_ only and P-0.4 group inject 0.4 ml TSPP only). One hour after the subcutaneous injection, the treatment groups’ models were exposed to visible light (500–550 nm) for 30 minutes. PDT continued for 22 days. On day 23 all animals were euthanized for further sampling. Along with SD rats, DBA/1 mice were also selected for more obvious and detailed fluorescence imaging. DBA/1 mice were also divided into three groups, i.e. normal group without RA disease, control group with RA disease (TP-0) and treatment group against RA disease (TP-0.4).

### CIA induction

Standard protocols were followed as described earlier^45^,^46^. Briefly, an equal amount of Collagen type II and Freund’s adjuvant (1 mg ml^−1^) were mixed until white water insoluble emulsion was formed. Then immediately within one hour 0.2 ml (rats) and 0.05 ml (mice) of emulsion was injected subcutaneously at the base of tail. At the day 18–21 all the rats showed signs of arthritis. Moreover, a booster dose was used at day 18 when needed and immediate signs were apparent within three days.

### Arthritis Score (AS)

Before and after the injection of TP, the Arthritis Score was calculated by the scoring method as defined earlier^47^ starting from 0 to 4 i.e. 0 (no obvious swelling and erythema); 1 (erythema and obvious mild swelling in any foot joint); 2 (erythema and mild swelling involving multiple foot or feet joint(s)); 3 (erythema and moderate swelling involving multiple feet or foot joint(s) difficulty in movement); 4 (erythema and severe foot or feet swelling involving all joints, ankylosis and dragging of foot with severe lameness).

### Photosensitizer preparation and injection

Tetra Sulphonatophenyl Porphyrin (TSPP) (Fig.S2) was purchased from ABI Chemicals and TiO_2_ (Fig. S3) was provided by Dr. Xiao Hua Lu (College of Chemical Engineering, Nanjing University of Technology, Nanjing 210009 China). To prepare the TSPP-TiO_2_ nanomaterial, TSPP was dissolved in deionized ultrapure water to obtain 0.05 mg/ml concentration. The diameter of the porous TiO_2_ nanowhiskers was less than 100 nm, as shown in Fig. S3. We selected an excitation wavelengths of the nanocomposites of TSPP and TiO_2_ around 500 ~ 550 nm, the emission wavelengths of the composite is around 600 ~ 640 nm. The TSPP was loaded on TiO_2_ by the means of physical adsorption. We have tested the relevant drug loading (DL) and the results indicated that DL could be achieved at 15.7% when TSPP was 0.05 mg/ml and TiO_2_ was 0.5 mg/ml. During the *in vivo* study, TiO_2_ was also dissolved in ultrapure deionized water to obtain a concentration of 0.5 mg/ml. Then both TSPP and TiO_2_ were mixed together according to the velum ratio 1:1 to obtain TP (0.05 mg/ml TSPP + 0.5 mg/ml TiO_2_) of which was then injected 0.4 ml (rats) and 0.1 ml (mice) in TP-0.4 groups. For T-0.4 and P-0.4 groups, only 0.5 mg/ml TiO_2_ and 0.05 mg/ml TSPP were injected 0.4 ml (rats), respectively. The TP-0 group was kept as a control.

### Blood sampling and analysis

Blood was collected by 3 ml intarcardiac injection with 25 gauge needle size under standard operative protocols^48^. Serum was removed from blood by centrifugation and whole blood for complete blood cells count (CBC) was preserved in 2 ml EDTA tubes.

### Histopathology

Feet were collected from each animal and stored in 10% formalin for at least 24 hours and then decalcified in 15% EDTA for two weeks. Then after dehydration, standard paraffin embedding protocols were used to prepare 6 μm thick slides and stained with Eosin & Hematoxylin stain^49^.

### Cell culture and imaging

Fibroblast cells were obtained from the synovial membrane of rat CIA models. Then cultured in DMEM standard medium containing 10% FBS and 1% Penicillin-Streptomycin Solution at 37^o^ C temperature, 5% CO2 and 95% humidity^50^,^51^. Fibroblast cells were sub-cultured in six well plates and added 100 μl of TP solution. Then after 24 hours the TP treated cells were processed for standard confocal microscopy by preserving in 3.7% paraformaldehyde solution.

### ELISA

ELISA was performed on the serum samples of SD-rats to find the concentration of TNF-α and IL-17. Rat ELISA ready set go kit (R&D Systems, Inc.) was used for both TNF-α and IL-17 according to instructions provided by the manufacturer.

### Fluorescence imaging

The animals were anesthetized under general anesthesia^48^ and fur was removed from all the feet for better imaging. Then 0.4 ml (SD rats) and 0.1 ml (DBA/1 mice) of TP solution was injected intravenously and within 10 minutes imaging was acquired on Perkin Elmer animal imaging system (IVIS Lumina XRMS Series III, with excitation wavelength of 520 nm and emission wavelength of 620 nm). The ROI (regions of interest) analysis was measured with Perkin Elmer Image software. First fluorescence imaging of DBA/1 mice was done before the onset of clinical signs (day 16 of first Collagen-Adjuvant injection) and then repeated after onset of clinical signs (on day 28 of first Collagen-Adjuvant injection).

### Determination of singlet oxygen quantum yields

Singlet oxygen quantum yields were determined using a time-resolved Nd:YAG laser set-up (excitation at 532 nm, Minilase II, New Wave Research Inc.) and a liquid N_2_ cooled Ge photodetector (Applied Detector Corporation Model 403S). A Schott color glass filter (model RG850; cut-on 850 nm; Newport, USA) taped to the sapphire entrance of the detector and a long wave pass filter (silicon filter model 10 LWF ~ 1000; Newport, USA), and a band pass filter (model BP-1270–080-B*; CWL 1270 nm; Spectrogen, USA) were used to allow to filter out all radiation outside the NIR range. Signals were digitized on a LeCroy 9350 CM 500 MHz oscilloscope and analyzed using Origin software. All quantum yield measurements were carried out at ambient temperature and air.

### Data analysis

Data was initially stored in MS excel and statistical program SPSS version 18 was used for analysis of variance (ANOVA).

## Additional Information

**How to cite this article**: Zhao, C. *et al.* Bio-imaging and Photodynamic Therapy with Tetra Sulphonatophenyl Porphyrin (TSPP)-TiO_2_ Nanowhiskers: New Approaches in Rheumatoid Arthritis Theranostics. *Sci. Rep.* **5**, 11518; doi: 10.1038/srep11518 (2015).

## Supplementary Material

Supplementary Information

Supplementary Movie S1

Supplementary Movie S2
