# Supplementary material for: Bio-imaging and Photodynamic Therapy with Tetra Sulphonatophenyl Porphyrin (TSPP)-TiO2 Nanowhiskers: New Approaches in Rheumatoid Arthritis Theranostics
Source: Sci Rep. 2015 Jul 8;5:11518. doi: 10.1038/srep11518 (PMC4648397; doi:10.1038/srep11518)
Supplement: Supplementary Information [file srep11518-s1.doc]

**Title:** **Bio-imaging and** **Photodynamic Therapy with Tetra Sulphonatophenyl Porphyrin (TSPP)-TiO_2_ Nanowhiskers: New Approaches in Rheumatoid Arthritis Theranostics**

Chunqiu Zhao^1, ‡^, Fawad Ur Rehman^1, ‡^, Yanlong Yang^3^, Xiaoqi Li^4^, Dong Zhang^2^, Hui Jiang^1^, Matthias Selke^2^, Chongyang Liu^3^, Xuemei Wang^1^,*

^1^State Key Laboratory of Bioelectronics, School of Biological Science and Medical Engineering, Southeast University, Nanjing 210096, China.

^2^Department of Chemistry and Biochemistry, California State University, Los Angeles, CA 90032, USA

^3^Daping Hospital, Third Military Medical University, Chongqing, 400042 (P.R. China)

^4^NanJing Foreign Language School, Nanjing 210096, China.

*Correspondence E-mail: X Wang: [xuewang@seu.edu.cn](mailto:xuewang@seu.edu.cn)

[‡] These authors contributed equally to this work.

**Supplementary Materials**

**Fig. S1 Determination of single oxygen quantum yields of TSPP and combination of TSPP and TiO_2_**

Plots of singlet oxygen NIR emission intensity (arbitrary units) vs. optical density at 532 nm for TSPP and TSPP-TiO_2_ (1:10) in D_2_O. The slopes were 0.175 and 0.121, respectively. TSPP-TiO_2_ (1:10).

**Fig. S2 Molecular structure of Tetra Sulphonatophenyl Porphyrin (TSPP)**


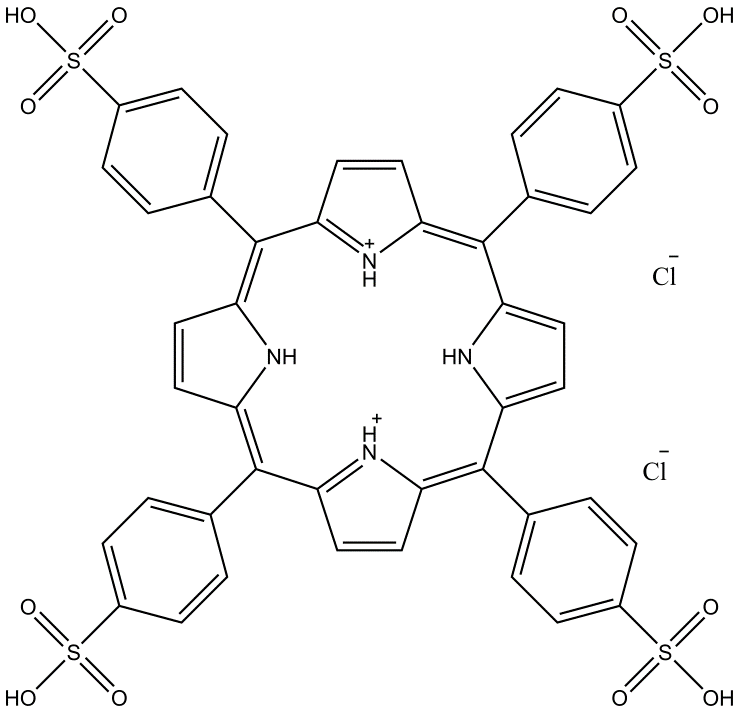


**Fig. S3 Morphology characterization of Titanium Dioxide (TiO_2_) nanowhiskers**


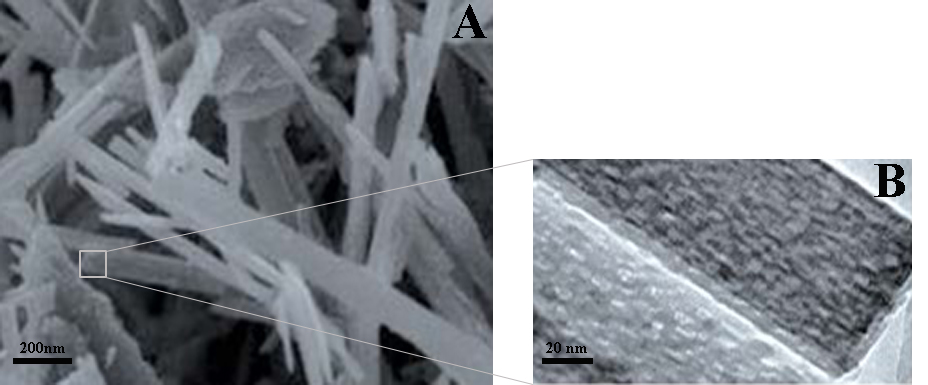


Characterization of TiO_2_ whiskers (A) SEM image (left) and (B) Typical TEM micrograph of a region (right),

**Fig. S4 Organs of rats from different treated groups (TP-0, T-0.4, P-0.4 and TP-0.4)**

**
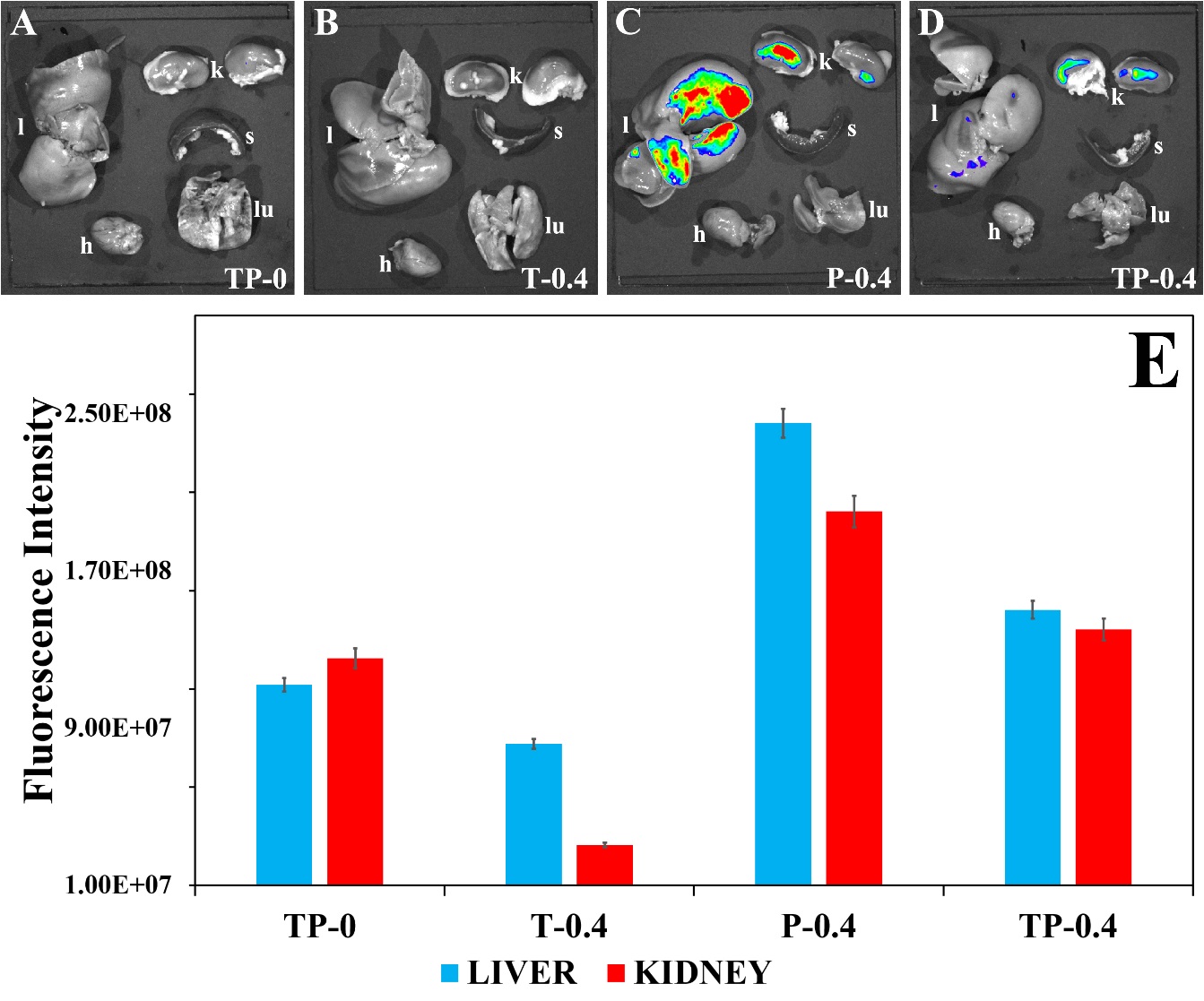
**

**A-D:** Fluorescence imaging of major organs (i.e., l for liver, h for heart, s for spleen, k for kidney and lu for lung) of control group with RA disease (TP-0), treatment group against RA disease (i.e., TP-0.4 group inject 0.4ml TiO2+TSPP compound, T-0.4 inject 0.4ml TiO2 only and P-0.4 inject 0.4ml TSPP only). **E:** The variations of fluorescence intensity in Liver and Kidney of different four groups.

**Movie S1 Movies of CIA SD-rat Pre Photodynamic therapy effect with TP solution**

Here in this clip the CIA model cannot move properly and drag its foot with severe lameness, prior to PDT with TSPP and TiO2 Solution. This model can be scored as (4) for RA.

**Movie S2 Movies of CIA SD-rat post Photodynamic therapy effect with TP solution**

In this clip on day 23 the severity of Lameness is almost subsided, no obvious inflammation and animal move more freely, can use both his feet and even can run.
